# The matricellular protein CCN5 prevents adverse atrial structural and electrical remodelling

**DOI:** 10.1111/jcmm.15789

**Published:** 2020-09-04

**Authors:** Min‐Ah Lee, Nour Raad, Min Ho Song, Jimeen Yoo, Miyoung Lee, Seung Pil Jang, Tae Hwan Kwak, Hyun Kook, Eun‐Kyoung Choi, Tae‐Joon Cha, Roger J. Hajjar, Dongtak Jeong, Woo Jin Park

**Affiliations:** ^1^ College of Life Sciences Gwangju Institute of Science and Technology Gwangju Korea; ^2^ Cardiovascular Research Center Icahn School of Medicine at Mount Sinai New York NY USA; ^3^ Bethphagen, S3‐203 Gwangju Institute of Science and Technology Gwangju Korea; ^4^ Basic Research Laboratory Chonnam National University Medical School Hwasun‐gun, Jeollanam‐do Korea; ^5^ Division of Cardiology Kosin University Gospel Hospital Busan Korea; ^6^ Phospholamban Foundation Amsterdam The Netherlands; ^7^ Department of Molecular and Life Science College of Science and Convergence Technology Hanyang University ERICA Campus Ansan Gyeonggi‐do Korea

**Keywords:** atrial fibrillation, atrial fibrosis, CaMKII, CCN5

## Abstract

Atrial structural remodelling including atrial hypertrophy and fibrosis is a key mediator of atrial fibrillation (AF). We previously demonstrated that the matricellular protein CCN5 elicits anti‐fibrotic and anti‐hypertrophic effects in left ventricles under pressure overload. We here determined the utility of CCN5 in ameliorating adverse atrial remodelling and arrhythmias in a murine model of angiotensin II (AngII) infusion. Advanced atrial structural remodelling was induced by AngII infusion in control mice and mice overexpressing CCN5 either through transgenesis (CCN5 Tg) or AAV9‐mediated gene transfer (AAV9‐CCN5). The mRNA levels of pro‐fibrotic and pro‐inflammatory genes were markedly up‐regulated by AngII infusion, which was significantly normalized by CCN5 overexpression. In vitro studies in isolated atrial fibroblasts demonstrated a marked reduction in AngII‐induced fibroblast trans‐differentiation in CCN5‐treated atria. Moreover, while AngII increased the expression of phosphorylated CaMKII and ryanodine receptor 2 levels in HL‐1 cells, these molecular features of AF were prevented by CCN5. Electrophysiological studies in ex vivo perfused hearts revealed a blunted susceptibility of the AAV9‐CCN5–treated hearts to rapid atrial pacing‐induced arrhythmias and concomitant reversal in AngII‐induced atrial action potential prolongation. These data demonstrate the utility of a gene transfer approach targeting CCN5 for reversal of adverse atrial structural and electrophysiological remodelling.

## 1 INTRODUCTION

Atrial fibrillation (AF), the most prevalent arrhythmia in humans, is often accompanied by heart failure (HF). This arrhythmia is associated with the increased risk of stroke, sudden death, and cardiovascular morbidity and mortality.^1^ Despite intensive studies spanning over 100 years, a clear understanding of AF mechanisms is still lacking, hampering the development of effective therapeutic approaches that are currently limited to destructive ablation techniques or sub‐optimal (and potentially pro‐arrhythmic) pharmacotherapy.^2^, ^3^, ^4^ While ablation is effective for treating paroxysmal AF episodes, its utility in the treatment of sustained AF arising in the setting of heart failure is inadequate. Instead, pharmacological approaches are used to achieve either rate or rhythm control. These approaches, however, are mired by extracardiac toxicities and pro‐arrhythmic effects, including risk of torsade de pointes and sudden death.

Adverse atrial structural and electrical remodelling are believed to form both the substrate and triggers required for AF initiation and maintenance. Classic anti‐arrhythmic approaches targeting individual ion channels have largely failed to prevent AF in structurally remodelled hearts, pointing to the need to address the structural abnormalities that promote AF, namely interstitial fibrosis, myocyte hypertrophy and myolysis. Of particular importance to the maintenance of AF is the development of interstitial atrial fibrosis that ultimately disrupts cell‐to‐cell electrical conduction and triggers single/multiple‐circuit re‐entry.^5^, ^6^ Fibroblast to myofibroblast trans‐differentiation is the key mechanism underlying pro‐fibrotic structural remodelling.^7^ In addition, defective Ca^2+^ handling is widely believed to mediate the arrhythmic triggers required for AF initiation.^8^, ^9^ Specifically, abnormal diastolic Ca^2+^ leak from the sarcoplasmic reticulum (SR) creates a transient inward current (I_ti_) that produces delayed afterdepolarizations (DADs). In a mouse model, inhibition of Ca^2+^‐ and calmodulin‐dependent protein kinase II (CaMKII) suppresses hyper‐phosphorylation of ryanodine receptor 2 (RyR2) at Ser2814 and aberrant SR Ca^2+^ leak.^10^

CCN5 is a member of the CCN family of matricellular proteins (CCN1‐6) that shares conserved modular domains, including (1) insulin‐like growth factor‐biding domain, (2) von Willebrand factor type‐C domain, (3) thrombospondin type‐1 domain and (4) C‐terminal cysteine‐knot domain.^11^ CCN5 is indeed a structurally unique protein that lacks the cysteine‐rich C‐terminal domain present in other CCN family members. Secreted and matrix‐associated CCN5 regulates diverse aspects of cell function, including signalling, adhesion, migration and proliferation.^12^ In previous studies, we identified potent anti‐hypertrophic and anti‐fibrotic properties of CCN5 in the ventricles of mice that underwent transverse aortic constriction.^13^ We further showed that CCN5 overexpression following TAC reverses pre‐established ventricular fibrosis by promoting myofibroblast‐specific apoptosis.^7^

In the present study, the putative role of CCN5 in either preventing or treating adverse atrial remodelling and atrial arrhythmias was investigated in CCN5 overexpressing transgenic (CCN5 Tg) mice as well as mice treated with AAV9‐mediated CCN5 gene delivery. We also utilized angiotensin II (AngII) to induce AF in mice. Activation of renin‐angiotensin system is thought to play a key role in the development of AF in humans.^14^ Accordingly, AngII is widely used to recapitulate the pathogenic scenarios of AF in animal models. Our data demonstrate that CCN5 attenuates AngII‐induced atrial interstitial fibrosis, atrial electrical remodelling and atrial arrhythmia. Moreover, CCN5 modulates phosphorylation of CaMKII and RyR2 in atrial myocytes in vitro and inhibits trans‐differentiation of atrial fibroblast into myofibroblast in vitro. Therefore, these data suggest that CCN5 may be a viable target for the treatment of AF arising in the setting of structural heart disease.

## 2 MATERIAL AND METHODS

### 2.1 Animals

All experimental procedures were approved by the Animal Care Committee of the Gwangju Institute of Science and Technology (Approval number: GIST‐2017‐028) and the Institutional Animal Care and Use Committee of the Icahn School of Medicine at Mount Sinai (Protocol number: IACUC‐2017‐0200). CCN5 Tg mice were generated as described previously.^13^ Male C57Bl/6 WT and CCN5 Tg mice (8‐10 weeks old, 20‐25 g bodyweight) underwent angiotensin II (AngII) infusion to promote adverse atrial remodelling. An osmotic mini‐pump (Alzet 1002, Alzet) was implanted subcutaneously for constant infusion of AngII (3 mg/kg/day) for 2 weeks as described previously.^15^

### 2.2 Histological studies

Mice were killed 14 days after AngII infusion. The hearts were arrested at end‐diastole, and the left atria were excised and weighed separately. Isolated atria were fixed in 10% formalin, embedded in paraffin and cut into 5 μM sections. To measure the fibrotic area, Masson's trichrome staining was performed. The fibrotic areas were stained with blue, and the normal tissue was stained with red. The fibrotic area was calculated by the ratio of the total area of fibrosis to the total area of the section using Aperio Imagescope (Leica Biosystems).

### 2.3 Quantitative real‐time (QRT)‐PCR

Total RNA was isolated from atria using TRI reagent (Molecular Research Center). Reverse transcription was performed using ImpromII reverse‐transcriptase (Promega) with oligo‐dT priming. PCR was performed using a Thermal Cycle Dice Real Time System TP800 (Takara) with SYBR Green (Takara) as a fluorescent dye. The primers used in this study are shown in Table S1.

### 2.4 HL‐1 cell culture

HL‐1 cells were kindly provided by Dr Steve Cho (GIST, Korea) and cultured in supplemented Claycomb medium (Sigma‐Aldrich) containing 10% foetal bovine serum (FBS; HyClone), 100 U/ml penicillin/streptomycin, 0.1 mM Norepinephrine (Sigma‐Aldrich) and 2 mM L‐Glutamine at 37°C in 5% CO_2_ contained incubator. Cells were cultured on dishes coated with 0.02% gelatin and 12.5 μg/ml fibronectin. The medium was changed every day.

### 2.5 Western blot analysis

Atrium cell lysates were obtained by solubilizing with RIPA buffer (0.1% SDS, 50 mM Tris‐HCl (pH 7.4), 150 mM NaCl, 1% NP‐40, 0.5% sodium deoxycholate; Boston BioProducts) and protease inhibitor cocktail (Boehringer Mannheim, Germany). Total cell lysates were separated by SDS‐PAGE and transferred to a PVDF membrane (Schleicher & Schuell, Germany). The membrane was blocked with 5% non‐fat milk and incubated with antibodies against phosphorylated CaMKII (Thr287) (Thermo), CaMKII (Santa Cruz), phosphorylated RyR2(Ser2814) (Badrilla), phosphorylated RyR2 (Ser2808) (Badrilla), RyR2 (Santa Cruz), Calsequestrin‐2 (Santa Cruz), NCX1 (LS Bio), α‐SMA (Sigma‐Aldrich), Collagen I (Rockland), TGF‐β1 (Santa Cruz), α‐tubulin (Santa Cruz), GAPDH (Laboratory made) and CCN5 (Genscript). Incubation with the primary antibody was usually carried out overnight in a cold room. The membrane was incubated with a secondary antibody conjugated to horseradish peroxidase (HRP) (Jackson Immuno‐research) and developed using a chemiluminescent substrate (Dozen). When necessary, the values for protein expression levels were obtained by normalization to the expression levels of GAPDH.

### 2.6 Production of conditioned medium

For analysis of CCN5 functions in vitro, CCN5‐containing conditioned medium (CM‐CCN5) was prepared by transiently transfecting HEK293 cells with pcDNA‐CCN5‐HA plasmids. This plasmid was overexpressed in HEK293 cells using Lipofectamin 2000 according to the manufacturer's instructions (Invitrogen). Cells were subsequently incubated for 24 hours before supernatants were collected by centrifugation at 220 x *g* for 1 min.

### 2.7 Isolation and culture of rat left‐atrial fibroblasts

Adult left‐atrial fibroblasts were isolated from the hearts of Sprague‐Dawley (SD) rats. The left atrium was minced, pooled and digested in a collagenase solution as previously described.^16^ Left‐atrial fibroblasts were pelleted at 220 x *g* for 10 min and resuspended in DMEM supplemented with 5% foetal bovine serum and 1% antibiotics. After 2‐3 days in culture, confluent preparations of fibroblasts were treated with 100 nM AngII and CM‐CCN5 for 48 hr. Left‐atrial fibroblasts were used as primary passage for experiments.

### 2.8 Immunocytochemistry analysis

Cells were seeded at a density of 1000 cells per 16‐mm coverslip, fixed with 4% paraformaldehyde, permeabilized with 0.5% Triton X‐100 and blocked with 5% BSA. Cells were incubated with antibody against α‐SMA (Sigma‐Aldrich). Nuclei were stained with Hoechst dye. Immunofluorescence was analysed by a Fluoview FV 1000 confocal laser scanning microscope.

### 2.9 Adeno‐associated viruses (AAV)

Generation of AAV expressing CCN5, AAV9‐CCN5, was described in our previous work.^7^ AAV9‐VLP (Virus‐like particle), viral particles that contain no viral genome,^17^ was used as a control virus throughout this study.

### 2.10 Optical mapping of langendorff‐perfused intact mouse hearts

8‐ to 10‐week‐old male C57Bl6/J mice underwent AngII infusion using an osmotic pump implanted for 2 weeks at a rate of 3 mg/Kg/day. Gene transfer of 5 × 10^11^ viral genome AAV9‐CCN5 or AAV9‐VLP (as control) was performed via tail vein injection. 4 weeks following gene transfer, mice were killed for detailed ex vivo electrophysiological (EP) assessment using optical action potential mapping. Hearts were stained with a voltage reporter dye (Di‐4‐ANEPPS, Invitrogen) and exposed to a monochromatic light (λ = 530nm, Mightex BioLED). Emitted light was filtered and projected onto a CCD camera (SciMeasure, SciMeasure Analytical Systems, USA) at a frame rate of 1 kHz. Volumetric electrocardiograms (vECG) were obtained continuously throughout the experiment for rhythm analysis (Biopac Systems MP150). Hearts were stimulated from the LA at progressively increasing frequencies at 2.5 × the diastolic threshold (DT), from 7 Hz (PCL 140ms) with increments of 2‐3 Hz until arrhythmia initiation. Optical capture was confirmed for every frequency of stimulation. The arrhythmia threshold was defined as the atrial pacing frequency at which arrhythmia was first induced and sustained (ie maintained >30 s). Data analysis was performed using custom‐developed software written in MATLAB (the MathWorks) that was optimized for the mouse heart. Spatiotemporal filtering by binning of 5 × 5 pixels was performed to increase the signal/noise ratio of the optical signal. Subsequently, activation times were selected using a 50% threshold and signal averaging from consecutive pacing cycles was used to construct representative activation maps. Due to signal instabilities at the low‐frequencies part of the signal, a threshold was set at 10% takeoff and 90% repolarization from the peak. To avoid including potential artefacts in our calculations, APD_50_ and APD_75_ were determined by the interval between the time point of the AP at the defined 10% threshold and the time at which the normalized AP repolarizes back to 50% and 75% from the peak, respectively. ADP_90_ was not determined as a reliable parameter due to significant reverberations of the signal at 90% repolarization, which can mislead interpretations.

### 2.11 Statistics

Statistical analyses were performed using Student's *t* test and one‐way ANOVA followed by post hoc Tukey test wherever appropriate (Prism 8, GraphPad). Significant differences are indicated by a single asterisk (*) (*P* < 0.05) or a double asterisk (**) (*P* < 0.01). Data in the figures represent the mean ± the SD.

## 3 RESULTS

### 3.1 CCN5 prevents AngII‐induced atrial fibrosis in CCN5 Tg mice

**FIGURE 1:**
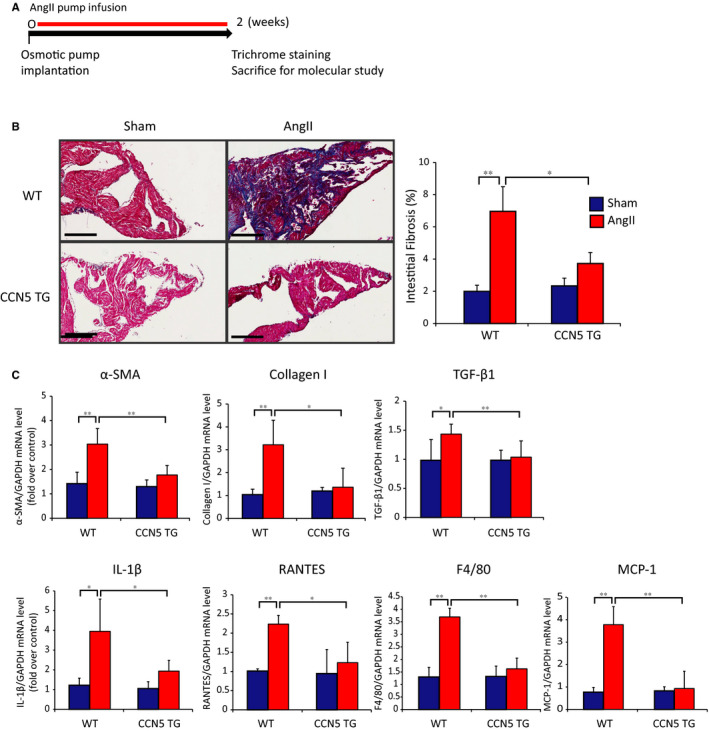
CCN5 prevents AngII‐induced atrial fibrosis in CCN5 Tg mice. A, Experimental scheme is shown for (B) and (C). Mice were sham‐operated or AngII‐infused osmotic pump transplantation for 2 weeks. B, Atria were sectioned and stained with Masson's trichrome. Representative images are shown (Left). The percentage of interstitial fibrotic areas is plotted (Right). Original magnification: 20 X, Scale bar: 200 μm. C, mRNA was isolated from left atrial and subjected to qRT‐PCR. Relative expression of pro‐fibrotic and pro‐inflammatory genes is shown. (n = 5‐6 per group). Error bar = SD, *P < 0.05, **P < 0.01

CCN5 Tg mice used in this study expressed CCN5 under the control of the cardiac muscle‐specific enhanced α‐myosin heavy chain promoter. To examine the effects of CCN5 on atrial fibrosis, AngII was administrated continuously over a 2‐week period using an osmotic pump. Hearts obtained from the experiments outlined in Figure 1A were subjected to histological analysis. Masson's trichrome staining of the left atrium revealed massive atrial fibrosis in Ang II‐infused WT but not CCN5 Tg mice (Figure 1B). Analysis with qRT‐PCR revealed that relative expression levels of pro‐fibrotic and pro‐inflammatory markers, including α‐smooth muscle actin (α‐SMA), collagen I, TGF‐β1, interleukin‐1β (IL‐1β), regulated upon activation normal T‐cell expressed and secreted (RANTES), F4/80, and monocyte chemoattractant protein‐1 (MCP‐1), were significantly greater in AngII‐infused WT compared to CCN5 Tg mice (Figure 1C). These data suggest that CCN5 overexpression ameliorates AngII‐induced atrial fibrosis.

### 3.2 CCN5 prevents AngII‐induced CaMKII activation in atrial myocytes and atrial fibroblast to myofibroblast trans‐differentiation

**FIGURE 2:**
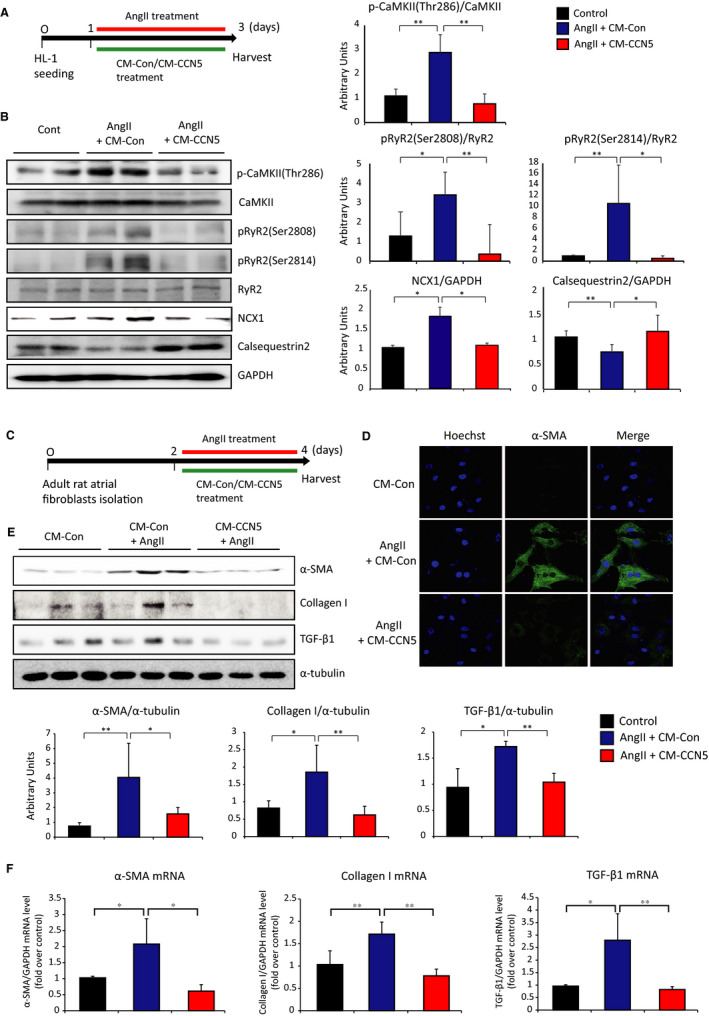
CCN5 prevents AngII‐induced CaMKII activation in atrial myocytes and atrial fibroblast to myofibroblast trans‐differentiation. A, Experimental scheme is shown for (b). HL‐1 cells, atrial myocyte cell line, were cultured in the presence of AngII and control conditioned media (CM‐Con) or CCN5 containing conditioned media (CM‐CCN5) for 48 hours. B, Total cell lysates from HL‐1 were immunoblotted with antibodies against phospho‐CaMKII (Thr286), total CaMKII, phospho‐RyR2 (Ser2808), phospho‐RyR2 (Ser2814), total RyR2, calsequestrin‐2, NCX1 and GAPDH. Quantified protein levels were plotted (Right). C, Experimental scheme is shown for D, E and F. Atrial fibroblasts were isolated from adult SD rats and cultured in the presence of AngII and CM‐Con or CM‐CCN5 for 48 hours. D, Immunofluorescence images of atrial fibroblasts were stained with anti‐α‐SMA antibody and Hoechst dye. Original magnification: 60 Χ. E, Total cell lysates from atrial fibroblasts were immunoblotted with antibodies against α‐SMA, collagen I, TGF‐β1 and α‐tubulin. Quantified protein levels are plotted (Bottom). F, mRNA was isolated from atrial fibroblasts and subjected to qRT‐PCR. Relative expression of pro‐fibrotic genes is presented. (n = 4‐6 per group). Error bar = SD *P < 0.05, **P < 0.01

Stress‐induced CaMKII hyper‐activation has been shown to play a critical role in the pathophysiology of AF and HF.^18^ To determine the extent of CaMKII activation by AngII, we used a mouse atrial cardiomyocyte cell line, HL‐1. All in vitro experiments for CCN5 function were performed using CCN5‐containing conditioned medium (CM‐CCN5) (Figure 2A). Treatment of HL‐1 cells with AngII in the presence of control conditioned medium (CM‐Con) resulted in increased phosphorylation of CaMKII, RyR2 (Ser2808 and Ser2814) and the expression of sodium/calcium exchanger 1 (NCX1) as assessed by Western blotting. The HL‐1 cells treated with AngII in the presence of CM‐CCN5 showed that phosphorylation of CaMKII and RyR2 (Ser2808 and Ser2814) was markedly reduced, and the level of NCX1 was down‐regulated. The expression of calsequestrin‐2, a key SR calcium‐binding protein, was significantly increased by CM‐CCN5 (Figure 2B).

Atrial interstitial fibrosis is a hallmark of adverse structural remodelling that promotes the perpetuation of atrial arrhythmias. To test the putative atrial anti‐fibrotic effect of CCN5, atrial fibroblasts were isolated and incubated with AngII either in the absence or in the presence of CCN5 in the medium (Figure 2C). While control fibroblasts exhibited markedly increased α‐SMA expression in response to AngII exposure, as shown by immunocytochemistry, co‐incubation with CCN5 fully prevented the AngII‐induced increase in α‐SMA expression (Figure 2D). Western blotting revealed that treatment with AngII significantly increased the expression of myofibroblast markers, α‐SMA, collagen I and TGF‐β1, which were diminished by co‐treatment with CM‐CCN5 (Figure 2E). The mRNA levels of myofibroblast markers were significantly increased in atrial fibroblasts, and CM‐CCN5 treatment completely reversed this AngII‐induced effect (Figure 2F). These results indicated that CCN5 completely inhibits AngII‐mediated atrial fibroblasts to myofibroblasts trans‐differentiation in vitro.

### 3.3 CCN5 reverses AngII‐induced atrial fibrosis in AAV‐mediated CCN5‐overexpressed mice

**FIGURE 3:**
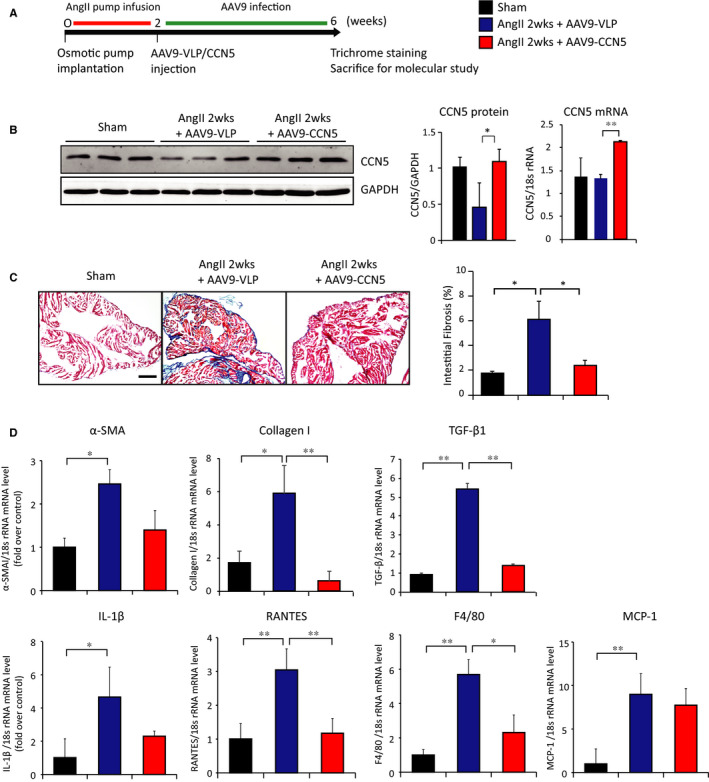
CCN5 reverses AngII‐induced atrial fibrosis in AAV9‐mediated CCN5 overexpressed mice. A, Experimental scheme is shown for B, C and D. Mice were underwent AngII‐infused osmotic pump implantation for 2 weeks. AAV9‐VLP or AAV9‐CCN5 (1 × 10^11^ viral genomes per mouse) was injected via tail vein. 4 weeks later, hearts were analysed. (B) Total cell lysates from heart atrium were immunoblotted with antibodies against CCN5 and GAPDH (Left). Quantified protein level (Middle) and mRNA level (Right) of CCN5 are plotted. Heart was sectioned and stained with Masson's trichrome. Representative images are shown from left atrium. The percentage of interstitial fibrotic areas is plotted. Original magnification: 20 X, Scale bar: 200 μm (D) mRNA was isolated from left atrial and subjected to qRT‐PCR. Relative expression of pro‐fibrotic and pro‐inflammatory genes is shown. (n = 5 per group). Error bar =SD *P < 0.05, **P < 0.01

We next examined whether in vivo AAV9‐mediated CCN5 delivery following 2 weeks of AngII infusion could reverse, or at least blunt, the proliferation of atrial fibrosis. Mice were subjected to osmotic pump implantation for chronic AngII infusion for 2 weeks to induce atrial structural remodelling. AAV9 carrying a control virus (AAV9‐VLP) or CCN5 (AAV9‐CCN5) was injected via tail vein and were analysed 4 weeks later (Figure 3A). Atrial CCN5 up‐regulation at the protein and mRNA levels by AAV9‐CCN5 was confirmed (Figure 3B). Hearts were excised and examined by Masson's trichrome staining for quantitation of atrial fibrosis. As shown, the extent of atrial fibrosis was indeed significantly increased after 2 weeks of AngII stimulation in mice that received AAV9‐VLP. In sharp contrast, AAV9‐CCN5–treated mice exhibited markedly attenuated fibrosis under the same conditions (Figure 3C). qRT‐PCR revealed that AngII induced the expression of pro‐fibrosis and pro‐inflammatory genes in AAV9‐VLP but not AAV9‐CCN5–treated mice (Figure 3D). These results indicate that exogenous CCN5 gene transfer reverses atrial fibrosis caused by chronic AngII infusion.

### 3.4 CCN5 suppresses atrial‐induced arrhythmia in AngII‐infused hearts by normalizing EP parameters

**FIGURE 4:**
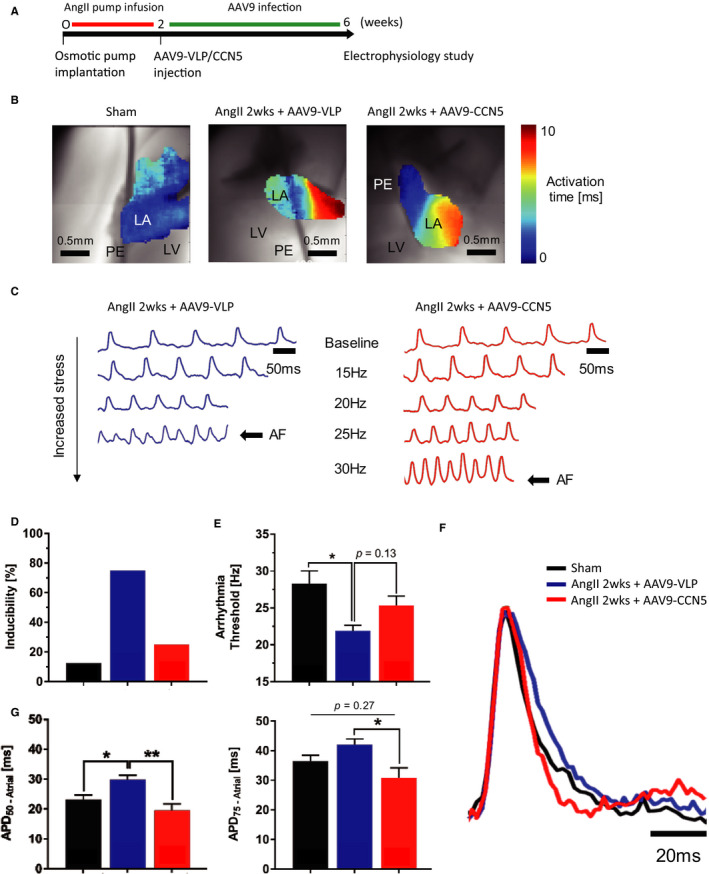
CCN5 decreases predilection for atrial‐induced arrhythmia in fibrotic hearts. A, Experimental scheme is shown. B, Representative maps for each group showing delays in atrial activation time at baseline (10 Hz) in AngII hearts (7.6 ± 2.4 ms) compared to sham (4.5 ± 1.6 ms) indicative of conduction slowing, which was partially reversed in AAV9‐CCN5–treated hearts (5.8 ± 1.5 ms). Scale bar: 0.5 mm, activation time: 0‐10 ms, LV = left ventricle, LA = left atrium, PE = pacing electrode. C, Representative optical mapping traces showing that a higher pacing frequency is necessary to induce atrial arrhythmia in AAV9‐CCN5–treated hearts compared to AngII, indicating lower arrhythmia vulnerability and reverse remodelling. Scale bar: 50 ms AF = atrial fibrillation. D, Bar graph shows a higher susceptibility to atrial arrhythmia at cut‐off frequency of 22 Hz (PCL 44 ms). E, Bar graph shows a higher susceptibility to atrial arrhythmia at cut‐off frequency of 22 Hz (PCL 44 ms). E, Bar graph shows a significantly lower threshold to induced atrial arrhythmia in the AngII hearts (21.9 ± 0.8 Hz in AngII, 28.3 ± 1.7 Hz in sham) *P < 0.05 (one‐way ANOVA followed by post hoc Tukey test). F and G, Representative atrial action potential and APD bar graphs at 10 Hz showing a prolongation of APD in fibrotic hearts (APD_50_ = 29.9 ± 1.4 ms in AngII vs. APD_50_ = 23.1 ± 1.5 ms in sham). AAV9‐CCN5 restores APD to normal values (APD_50_ = 19.5 ± 2.2 ms, APD_75_ = 30.9 ± 3.4 ms in AAV9‐CCN5 vs. APD_75_ = 42.1 ± 1.9 ms in AngII). Scale bar 20 ms *P < 0.05, **P < 0.01 (one‐way ANOVA followed by post hoc Tukey test). Data obtained from eight mice were analysed through D ~ G

We next examined whether CCN5 altered AngII‐induced atrial electrophysiological properties and the propensity to atrial arrhythmias induced by rapid atrial pacing in ex vivo perfused hearts from AAV9‐VLP and AAV9‐CCN5–treated mice relative to sham (Figure 4A). Arrhythmia propensity was determined by first comparing the incidence of sustained episodes (>30 seconds) of atrial arrhythmia in each group at a cut‐off atrial pacing frequency of 22 Hz. We subsequently compared the atrial pacing frequency threshold that was required for the genesis of sustained atrial arrhythmias in each heart. Atrial arrhythmias were detected in real time using continuous monitoring of electrogram recordings before, during and after challenge with rapid pacing and were verified once induced using high‐resolution optical action potential mapping (Figure 4B and C). At the cut‐off pacing frequency of 22 Hz, sustained atrial pacing‐induced arrhythmia was generated in 75% of AAV9‐VLP–treated AngII hearts compared to only 25% of AAV9‐CCN5–treated AngII and 12.5% of sham hearts (Figure 4D, Table S2). Furthermore, quantification of the atrial pacing frequency threshold required for induction of sustained atrial arrhythmia in each heart revealed a strong trend towards reduced levels (ie increased vulnerability) in AAV9‐VLP (21.9 ± 0.8 Hz) compared to sham (28.3 ± 1.7 Hz, *P *< 0.05) and partial restoration by AAV9‐CCN5 treatment (25.4 ± 1.3 Hz, *P* = 0.13) (Figure 4E). We next examined the extent of atrial electrical remodelling as indexed by action potential decay (APD) prolongation. As expected, AngII infusion caused significant prolongation in APD_50_ and a trend towards longer APD_75_ compared to sham hearts (Figure 4F and G). Remarkably, CCN5 treatment reversed AngII‐mediated APD prolongation consistent with reversal of pathological atrial electrical remodelling (Figure 4F and G).

## 4 DISCUSSION

AF, the most prevalent rhythm disorder, is a major global public health epidemic that is increasing in both incidence and prevalence.^19^, ^20^ Structural remodelling, including increased fibrosis, is thought to contribute to the pathogenesis of AF by altering myocardial electrophysiological properties.^21^ We previously reported that the matricellular protein CCN5 elicits cardioprotective signalling that prevents the formation of massive ventricular fibrosis in response to chronic pressure overload. Remarkably, CCN5 delivery following the pro‐fibrotic insult was found to reverse pre‐established ventricular fibrosis by selectively promoting apoptosis in myofibroblasts, but not in cardiomyocytes or fibroblasts.^7^ Based on our previously reported findings of potent anti‐fibrotic effect of CCN5 in the stressed ventricle, we surmised that CCN5 may have a significant comparable role in preventing or combating adverse atrial structural and electrical remodelling.

Here, we demonstrate for the first time that CCN5 overexpression, either by transgenesis or by gene transfer, prevents AngII‐induced atrial remodelling, including atrial extracellular collagen deposition and reverses the expression of fibrosis‐related genes (Figures 1 and 3). In addition, electrophysiological studies revealed a reduction in the incidence of rapid atrial pacing‐induced arrhythmias by AAV9‐mediated CCN5 gene transfer (Figure 4C) and in CCN5 Tg mice (Figure S1).

CaMKII is a multifunctional serine‐threonine kinase that regulates myocardial stress responses. AF‐related DADs are caused by oxidation or/and phosphorylation of CaMKII leading to RyR2 phosphorylation at Ser2814. This, in turn, increases RyR2‐mediated SR Ca^2+^ leak.^22^ This diastolic Ca^2+^ leak from the SR through dysfunction of RyR2 is important in the pathogenesis of arrhythmia and heart failure.^23^ While CaMKII is normally autoinhibited, increased expression of its phosphorylated form in response to chronic AngII activation promotes AF.^24^ Thus, CaMKII may have a critical role in increased SR Ca^2+^ leak in AF. In this study, we found that AngII‐mediated increases in CaMKII and RyR2 phosphorylation in HL‐1 cells are prevented by co‐incubation with CCN5 (Figure 2B). Our finding suggests potent regulation of CaMKII phosphorylation by CCN5 in response to AngII stimulation, which leads to inhibition of abnormal SR Ca^2+^ leak. We previously showed that CCN5 counteracts CCN2 (aka CTGF) in the hearts.^13^ It was also shown that CCN2 binds to and activates tyrosine receptor kinases, which leads to activation of CaMKII and other signalling molecules.^25^ Therefore, it is likely that CCN5 inhibits the CaMKII phosphorylation indirectly through down‐regulating CCN2. This hypothesis remains to be evaluated.

Structural remodelling of the heart in AF is primarily associated with cardiac fibrosis, which disrupts myocardial electrophysiological properties, hinders normal atrial activation, and promotes the formation of re‐entrant circuits underlying AF.^26^ In the unstressed normal myocardium, cardiac fibroblasts do not adversely affect myocardial electrophysiological properties. On the other hand, in response to chronic stress, fibroblast proliferation and trans‐differentiation to activated myofibroblasts are thought to promote arrhythmias by direct hetero‐cellular (myocyte‐myofibroblast) coupling and/or paracrine signalling, including the release of key cytokines, including TGF‐β and IL‐1β. The activation of myofibroblasts, a key step during the generation of cardiac fibrosis, has been shown to be caused by the induction of Ca^2+^ through TRPM7 and TRPC3, which are Ca^2+^ permeable non‐selective cation channels.^27^, ^28^ Ca^2+^‐influx through TRP channels promotes CaMKII activation and induces ECM protein production.^29^ Thus, CCN5 could potentially regulate CaMKII activation in myofibroblasts. Further in‐depth studies will be required.

It appears that CCN5 acts as a transcriptional co‐activator or co‐repressor depending on the physiological context. Therefore, numerous other targets of CCN5 might also be involved in the beneficial role of CCN5 in atria. For example, CCN5 was shown to down‐regulate the expression of TGF‐β Receptor II as a transcriptional co‐repressor.^30^ Further elucidation of other targets of CCN5 is currently undergoing in our laboratory.

Taken together, this study suggests that CCN5 may provide a new target for the treatment of adverse atrial remodelling. One important question is how to normalize the CCN5 level in diseased atria. Gene therapy approaches have recently been explored in pre‐clinical and clinical studies for heart diseases.^31^, ^32^ As shown in this study, therapeutic genes can be delivered to atria by intravenous injection of recombinant AAV serotype 9. However, many challenges remain to be resolved before gene therapy approaches become clinically viable options for AF treatment.

Limitations: The mouse provides a reliable model to study and characterize electrical disturbances in the heart. However, it remains pivotal to recognize the differences between the murine and the human heart, which could limit the interpretations of the data acquired from the mouse.^33^ Therefore, the data presented here cannot be directly compared to the human substrate, where the conditions facilitating AF induction clinically were not completely addressed in this study. In this work, we focused on fibrosis as an essential substrate for arrhythmia induction,^21^ and we showed that additional stress such as tachycardia‐pacing helped unmask this predilection in our model. These findings should be interpreted with caution, since we did not consider other known cardiovascular risk factors that could play a role in long‐term remodelling, which probably play a more significant role in the clinical setting.

## CONFLICT OF INTERESTS

THK and WJP have co‐ownership interest in BethphaGen. The other authors have declared that no conflict of interest exists.

## AUTHOR CONTRIBUTIONS

WJP and D.J: Conceptualization and supervision of this work. M.‐AL, NR, MHS, JY, ML, SPJ, THK, HK, E.‐KC, T.‐JC, RJH, DJ and WJP: Research design, experiments and data analysis. M.‐AL, NR, MHS, D.J and WJP: Writing, revision, and final revision of the manuscript. Min‐Ah Lee: Data curation (equal); Formal analysis (equal); Validation (equal); Visualization (equal); Writing‐original draft (equal); Writing‐review & editing (equal). Nour Raad: Data curation (equal); Formal analysis (equal); Validation (equal); Visualization (equal); Writing‐original draft (equal); Writing‐review & editing (equal). Min Ho Song: Data curation (equal); Formal analysis (equal); Validation (equal); Visualization (equal); Writing‐original draft (equal); Writing‐review & editing (equal). Jimeen Yoo: Data curation (supporting); Formal analysis (supporting). Miyoung Lee: Data curation (supporting); Formal analysis (supporting). Seung Pil Seung Pil Jang: Data curation (supporting); Formal analysis (supporting). Tae Hwan Kwak: Data curation (supporting); Formal analysis (supporting). Hyun Kook: Data curation (supporting); Formal analysis (supporting). Eun‐Kyoung Choi: Data curation (supporting); Formal analysis (supporting). Tae‐Joon Cha: Data curation (supporting); Formal analysis (supporting). Roger J. Hajjar: Data curation (supporting); Formal analysis (supporting). Dongtak Jeong: Conceptualization (equal); Data curation (equal); Formal analysis (equal); Funding acquisition (equal); Supervision (equal); Validation (equal); Visualization (equal); Writing‐original draft (equal); Writing‐review & editing (equal). Woo Jin Park: Conceptualization (lead); Data curation (lead); Formal analysis (lead); Funding acquisition (lead); Supervision (lead); Validation (lead); Visualization (lead); Writing‐original draft (lead); Writing‐review & editing (lead).

## Supporting information

Supplementary Material

## Data Availability

I confirm that my article contains a Data Availability Statement even if no data are available (list of sample statements) unless my article type does not require one (eg Editorials, Corrections, Book Reviews, etc).
