# Supplementary material for: The matricellular protein CCN5 prevents adverse atrial structural and electrical remodelling
Source: J Cell Mol Med. 2020 Sep 4;24(20):11768–78. doi: 10.1111/jcmm.15789 (PMC7579720; doi:10.1111/jcmm.15789)
Supplement: Supplementary file 1 — Supplementary Material [file JCMM-24-11768-s001.docx]

**The matricellular protein CCN5 prevents adverse atrial structural and electrical remodeling**

Min-Ah Lee, PhD, Nour Raad, MD, PhD, Min Ho Song, BS, Jimeen Yoo, MS, Miyoung Lee, BS, Seung Pil Jang, PhD, Tae Hwan Kwak, MS, Hyun Kook, MD, PhD, Eun-Kyoung Choi, MS, Tae-Joon Cha, MD, PhD, Roger J. Hajjar, MD, Dongtak Jeong, PhD, Woo Jin Park, PhD


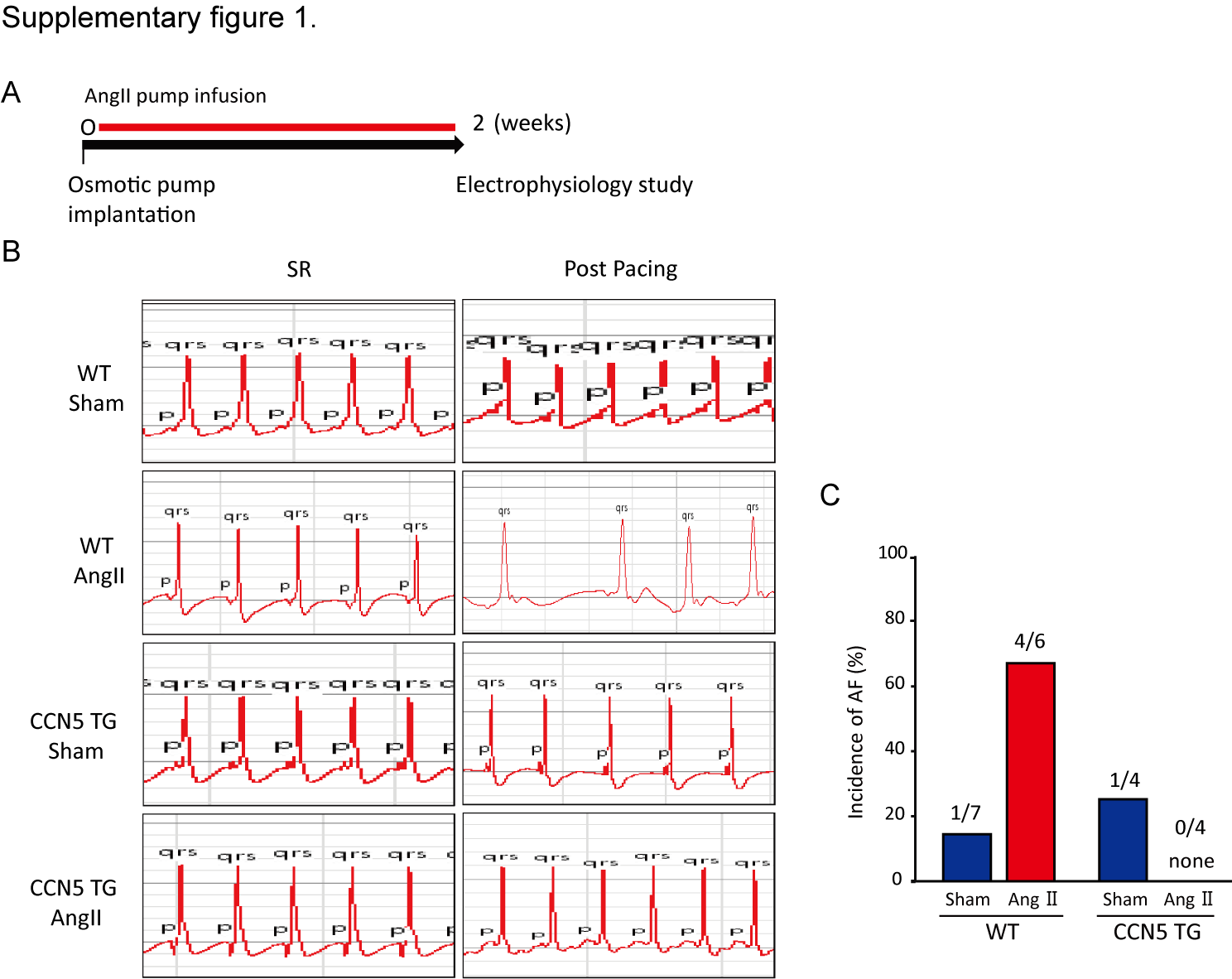


**Supplementary figure 1. CCN5 prevents AngII-induced AF phenotype in CCN5 Tg mice.**

(A) Experimental scheme is shown for B and C. Mice were sham-operated or AngII infused osmotic pump transplantation for 2weeks. Heart were performed by electrophysiological study using a Langendorff system. (B) Representative electrocardiogram (ECG) records before and after the burst pacing are presented. (C) Bar graph shows the incidence of atrial fibrillation phenotype.

**Supplementary table 1. Quantitative RT-PCR primer information**

| **Gene** | **Sequence** |
| --- | --- |
| Mouse α-SMA | 5’- CCCACCCAGAGTGGAGAA -3′  5′- ACATAGCTGGAGCAGCGTCT -3′ |
| Mouse Collagen I | 5′- CATGTTCAGCTTTGTGGACCT-3′  5′- GACGCTGACTTCAGGGATGT -3′ |
| Mouse TGF-β1 | 5′- TGGAGCAACATGTGGAACTC -3′  5′- CAGCAGCCGGTTACCAAG -3′ |
| Mouse IL-1β | 5′- TCCAGGATGAGGACATGATGAGCA -3′  5′- GAACGTCACACACACCAGCAGGTTA -3′ |
| Mouse RANTES | 5′- TGCAGAGGACTCTGAGACAGC -3′  5′- GAGTGGTGTCCGAGCCATA -3′ |
| Mouse F4/80 | 5′- CCTGGACGAATCCTGTGAAG -3′  5′- GGTGGGACCACAGAGAGTTG -3′ |
| Mouse MCP-1 | 5′- CATCCACGTGTTGGCTCA -3′  5′- GATCATCTTGCTGGTGAATGAGT -3′ |
| Mouse CCN5 | 5′- ATACAGGTGCCAGGAAGGTG -3′  5′- GTTGGATACTCGGGTGGCTA -3′ |
| Mouse GAPDH | 5′- CTCATGACCACAGTCCATGC -3′  5′- TTCAGCTCTGGGATGACCTT -3′ |
| Mouse 18s rRNA | 5′- GTAACCCGTTGAACCCCATT -3′  5′- CCATCCAATCGGTAGTAGCG -3′ |
| Rat α-SMA | 5′- TCTGTCTCTAGCACACAACTGTGAATG -3′  5′- TTGACAGGCCAGGGCTAGAAGGG -3′ |
| Rat Collagen I | 5′- AATGCACTTTTGGTTTTTGGTCACGT -3′  5′- CAGCCCACTTTGCCCCAACCC -3′ |
| Rat TGF-β1 | 5′- TGTTCGCGCTCTCGGCAGTG -3′  5′- CGGATGGCCTCGATGCGCTT -3′ |
| Rat GAPDH | 5′- ACCCAGCCCAGCAAGGATACTG -3′  5′- ATTCGAGAGAAGGGAGGGCTCCC -3′ |

**Supplementary table 2. Contingency**

| Group | ≤ 22Hz  (Below threshold) | ≥ 22Hz  (Above threshold) | Total |
| --- | --- | --- | --- |
| Sham | 1/8 | 7/8 | 8/8 |
| AngII | 6/8 | 2/8 | 8/8 |
| CCN5 | 2/8 | 6/8 | 8/8 |
